# Supplementary material for: Asymmetric Synthesis of Quaternary Hydantoins via a Palladium-Catalyzed Aza-Heck Cyclization
Source: J Am Chem Soc. 2025 Nov 14;147(49):44692–8. doi: 10.1021/jacs.5c16022 (PMC12703750; doi:10.1021/jacs.5c16022)

DWatson_Temidayo_Idowu_061_250218180530 02/18/25 18:05:30

DWatson_Temidayo_Idowu_061_250218180530 #45 RT: 0.46 AV: 1 NL: 6.47E8
T: FTMS + p ESI Full ms [100.0000-1500.0000]

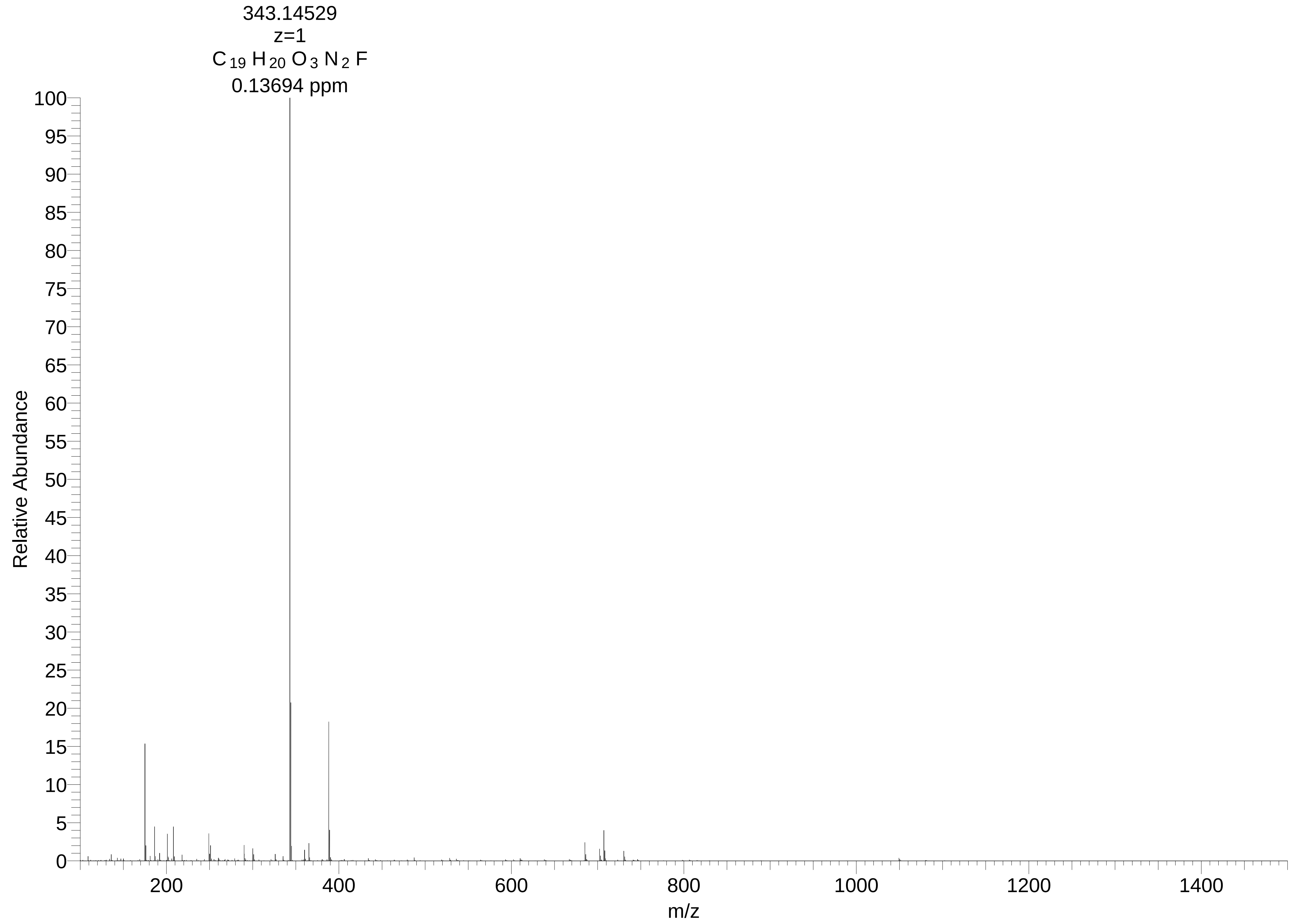

Supplement: Supplementary file 2 [file ja5c16022_si_002.zip › All NMR FID Files/S17/S17_AllNMR/HRMS/DWatson_TI_061.pdf]
